# Obstetric Tetanus in an Unvaccinated Woman After a Home Birth Delivery — Kentucky, 2016

**DOI:** 10.15585/mmwr.mm6611a7

**Published:** 2017-03-24

**Authors:** Anna Q. Yaffee, David L. Day, Glenda Bastin, Mary Powell, Sandra Melendez, Nancy Allen, Julie Miracle, Margaret Jones, Robert Brawley

**Affiliations:** ^1^Kentucky Department for Public Health; ^2^Epidemic Intelligence Service, CDC; ^3^Lincoln Trail District Health Department, Elizabethtown, Kentucky; ^4^Louisville Metro Public Health and Wellness, Louisville, Kentucky; ^5^Central Medical Associates, Elizabethtown, Kentucky.

On July 11, 2016, state and local health departments in Kentucky were notified of a case of obstetric tetanus in an unvaccinated woman. Obstetric tetanus, which occurs during pregnancy or within 6 weeks of the end of pregnancy, follows contamination of wounds with *Clostridium tetani* spores during pregnancy, or the use of contaminated tools or practices during nonsterile deliveries or abortions. CDC did not identify any cases of obstetric tetanus in the United States during 1972–2008 (*1*,*2*). State and local health departments in Kentucky investigated this case to identify risk factors and provide recommendations.

The patient, a woman aged 30 years, is a member of an Amish community. In late June, she delivered a child at home, assisted by an unlicensed community childbirth assistant. She had never received a vaccination for tetanus. Delivery was complicated by breech presentation, but no birth trauma, unsterile conditions, or other complications were reported. Nine days postpartum, the patient experienced facial numbness and neck pain, which progressed over 24 hours to stiff neck and jaw and difficulty swallowing and breathing. She was admitted to the hospital where a clinical diagnosis of tetanus was made, and 6,000 international units of tetanus immunoglobulin were administered intramuscularly. Endotracheal intubation and mechanical ventilation were required. Her hospital course was complicated by seizures and a need for prolonged respiratory support. After approximately a month, the patient was stable and discharged home.

The infant was monitored at home during the mother’s hospitalization. Tetanus immunoglobulin was recommended; however, the family declined treatment. A local advanced practice nurse performed weekly follow-up visits and noted no problems in the infant.

The close relationship between the local health department, health care providers, and the approximately 400-member Amish community facilitated contact with community leaders for an opportunity to discuss implementing Advisory Committee on Immunization Practices (ACIP) recommendations for tetanus immunization through a vaccination campaign. Door-to-door home visits in areas with vaccine-supportive community leaders were made by local health department staff members and the advanced practice nurse to explain the benefits of vaccination and provide vaccine. At the time of the campaign, there was one pregnant woman and one woman who was immediately postpartum in the community; both declined vaccination. Forty-seven (12%) persons were vaccinated, including 32 children aged ≤18 years. An age-appropriate diphtheria, tetanus, and pertussis vaccine (DTaP or Tdap) was administered to 30 (64%) of the 47 vaccine recipients. Because many community members reported having had pertussis disease and were opposed to receiving pertussis vaccine, 17 (36%) persons received age-appropriate tetanus and diphtheria toxoids without pertussis vaccine (DT or Td). Although none of the persons receiving vaccine had been previously vaccinated against any disease to date, none have agreed to complete the series because of little perceived ongoing vaccination need. Additional outreach initiatives are planned.

To prevent tetanus, ACIP recommends a 5-dose series of diphtheria and tetanus toxoids and acellular pertussis vaccine (DTaP) for children at ages 2, 4, 6, 15–18 months, and 4–6 years, followed by 1 dose of tetanus and diphtheria toxoids and acellular pertussis vaccine (Tdap) for adolescents aged 11–12 years. Previously vaccinated adults are recommended to receive routine booster doses of a tetanus-containing vaccine every 10 years, and unvaccinated adults should complete a 3-dose primary series (*3*,*4*). Pregnant women with unknown or incomplete tetanus vaccination histories should receive a series of 3 doses of tetanus and reduced diphtheria toxoids (Td) to protect against obstetric and neonatal tetanus (*5*). ACIP also recommends a dose of Tdap to all previously vaccinated pregnant women at 27 to 36 weeks’ gestation during each pregnancy, regardless of time of previous vaccination, to provide protection from pertussis to infants.

This case highlights the importance of tetanus vaccination for all persons as recommended by ACIP (*5*,*6*). Although Amish communities generally do not have religious objections to vaccination (*7*), preventive health care has not historically been accessed by this Amish community. Trust between the Amish community, local health department, and a familiar health care provider, as well as working within community members’ homes, and providing culturally appropriate education and recommendations through community leaders, facilitated vaccination of some persons. Ongoing outreach by health departments is beneficial to vulnerable, nonimmunized or underimmunized populations.
